# Full Factorial Comparison of the Diagnostic Performance of Three Nucleic Acid Extraction Kits and Three PRRSV RT-qPCR Assays Using Swine Oral Fluids of Known Status

**DOI:** 10.3390/microorganisms14020282

**Published:** 2026-01-26

**Authors:** Betsy Armenta-Leyva, Gaurav Rawal, Jianqiang Zhang, Berenice Munguía-Ramírez, Grzegorz Tarasiuk, Danyang Zhang, Rolf Rauh, Kyoung-Jin Yoon, Luis G. Giménez-Lirola, Jeffrey J. Zimmerman

**Affiliations:** 1 Department of Veterinary Diagnostic and Production Animal Medicine, College of Veterinary Medicine, Iowa State University, Ames, IA 50011, USA; betsyarl@iastate.edu (B.A.-L.); bmunguia@iastate.edu (B.M.-R.); luisggl@iastate.edu (L.G.G.-L.); 2 Department of Statistics, College of Liberal Arts and Sciences, Iowa State University, Ames, IA 50011, USA; 3 Tetracore Inc., Rockville, MD 20850, USA

**Keywords:** oral fluids, swine diagnostics, molecular assay evaluation, ECq, diagnostic sensitivity, diagnostic specificity, ROC analysis, efficiency, precision, reproducibility

## Abstract

Porcine reproductive and respiratory syndrome (PRRS) is one of the costliest diseases in swine production, causing >$1.2 billion USD in annual losses in the United States. Oral fluids are widely used for PRRS virus (PRRSV) surveillance, accounting for 42% of nearly 480,000 PRRSV RT-qPCR cases submitted to six Midwestern U.S. laboratories between 2020 and 2025. Despite this reliance, few studies have applied appropriate methodological approaches to compare the performance of commercial extraction and PRRSV RT-qPCR protocols for oral fluid specimens. In this study, we evaluated nine extraction-amplification protocols for PRRSV RNA detection, based on three commercial extraction kits and three commercial RT-qPCR assays. For each protocol, performance was evaluated using 314 oral fluid samples of known status (215 positive, 99 negative), collected under controlled conditions from 72 pigs assigned to five groups inoculated with contemporary PRRSV isolates and from one negative control group. The Cq values were normalized as efficiency-standardized Cqs (ECqs) and then analyzed by receiver operating characteristic (ROC) analysis. The mean amplification efficiencies ranged from 67 to 92%, repeatability from 0.98 to 0.99, and overall reproducibility was 0.91. The ROC AUCs ranged from 0.916 to 0.986, with significant pairwise differences (*p* < 0.05). At optimal ECq cutoffs, sensitivities ranged from 83 to 98.1% with 100% specificity. Normalization enabled objective protocol comparisons and statistically valid diagnostic cutoffs and supports future improvements in PRRSV diagnostics.

## 1. Introduction

Porcine reproductive and respiratory syndrome virus (PRRSV) remains the costliest disease in modern swine production. In the United States, for example, industry-wide losses have been estimated at over USD 1.2 billion annually [1]. In Europe, farm-level losses range from EUR 255 per sow in endemic settings [2] to over EUR 650,000 annually in severely affected wean-to-finish operations [3].

Given its substantial economic impact, accurate and timely detection of PRRSV in commercial herds is essential to its management and control. Thus, between 2020 and 2025, 479,743 diagnostic cases (the majority containing multiple samples for testing) were submitted to six Midwestern US veterinary diagnostic laboratories for PRRSV RT-qPCR testing [4]. The variety of specimens submitted for testing included blood-derived samples, environmental samples, oral fluid, processing fluid, semen, swabs (nasal, blood, oral), tissues, tongue tip fluids, and others. Among these, oral fluid samples were included in 42% of all submissions (199,179 cases). This reflects both the routine use of oral fluids in herd-level PRRSV surveillance and its integration into laboratory workflows and testing protocols.

In the laboratory, PRRSV detection has followed historical technological developments. Initially, PRRSV detection was based on isolation in either primary cells (macrophages) or continuous cell lines [5]. The introduction of PRRSV reverse transcription polymerase chain reaction (RT-PCR) technology precluded the need to isolate the virus from the clinical specimens [6,7,8,9]. By the early 2000s, real-time RT-PCR (RT-qPCR) assays provided a more efficient approach to PRRSV RNA detection [10,11]. After 2004, the availability of commercial PRRSV RT-qPCR assays facilitated standardized testing across laboratories [12].

A number of PRRSV commercial RT-qPCR assays are currently available and the test is used extensively. For example, the Iowa State University veterinary diagnostic laboratory performed approximately 1,400,000 PRRSV RT-qPCR tests between 2020 and 2025 [4]. Despite its widespread use, comparisons of diagnostic performance among commercial PRRSV RT-qPCRs, particularly for swine oral fluids, are limited. Moreover, the existing studies often lack appropriate methodological approaches for evaluating extraction and amplification workflows, thereby limiting the ability to validate and standardize diagnostic cutoffs. Therefore, the objective of this study was to apply a full-factorial design and the appropriate analytical methodology to evaluate the diagnostic performance of commercially available extraction and amplification protocols, using oral fluid samples from pigs inoculated with contemporary PRRSV isolates.

## 2. Materials and Methods

### 2.1. Experimental Design

Commercially available nucleic acid extraction kits (*n* = 3) and PRRSV RT-qPCR assays (*n* = 3) were evaluated in a 3 × 3 full factorial design (Table 1), using 99 PRRSV-negative and 215 PRRSV-positive pen-based oral fluid samples from five groups of pigs, each inoculated with a different contemporary PRRSV isolate, plus a negative control group [13]. Estimates of diagnostic sensitivity and specificity were determined by receiver operating characteristic (ROC) analysis.

### 2.2. PRRSV Isolates

All PRRSV-2 isolates used in this study were recovered from routine submissions to the Iowa State University (ISU) Veterinary Diagnostic Laboratory (Ames, IA, USA). Virus isolation, propagation, and titration were performed in ZMAC cells [14] to a concentration of 1 × 10^6^ TCID_50_ per ml, as previously described [15]. The pairwise nucleotide identity (%) between isolates, based on complete genome alignment, was determined by using the blastn suite-2sequences tool in NCBI BLAST^®^ (https://blast.ncbi.nlm.nih.gov/, accessed on 14 August 2025) [16] (Table 2).

### 2.3. Oral Fluid Samples from Pigs of Known PRRSV Infection Status

Pen-based oral fluid samples (*n* = 314) of known PRRSV status were derived from a study approved by the ISU Office of Research Ethics (IACUC-21-124; IBC-21-124) and conducted in biosafety level 2 (BSL-2) animal research facilities. Full details of this study are provided elsewhere [13].

Pigs (*n* = 72) in the study were serum tested for the PRRSV RNA and antibody 8 days prior to study enrollment. Upon arrival at 3 weeks of age, pigs were ear-tagged, re-tested for PRRSV infection, blocked by weight, and randomly assigned to one of 6 groups, i.e., 5 groups inoculated with PRRSV and a negative control group, using a random number generator (Excel Microsoft), and allowed to acclimate for 7 days. Groups to be inoculated with PRRSV were housed in separate rooms. Each room contained one large pen, which was divided into two smaller pens (inoculated pen and contact pen), using metal gating that allowed nose-to-nose interactions between pigs in adjacent pens. Initially, the inoculated pen was populated with 8 pigs and the contact pen remained vacant. Pigs were placed in the contact pen on day post-inoculation (DPI) 2.

Prior to inoculation, pigs were trained for oral fluid sampling, beginning 6 days before inoculation (designated as DPI −6). Training consisted of placing a 50 cm (19.7″) × 1.3 cm (1/2″) diameter, 3-strand, twisted 100% cotton rope (Skydog Rigging Supply, Lake in the Hills, IL, USA) on the pen floor for ~15 min and then hanging the rope from a bracket attached to the side of the pen gating. Thereafter, pen-based oral fluid samples were collected twice daily from DPI −5 to DPI 28. At the end of each study day, samples were aliquoted (10 aliquots per sample), labeled appropriately (including a unique random number), and stored at −80 °C until testing.

On DPI 0, pigs were inoculated with PRRSV isolates at a concentration of 1 × 10^6^ TCID_50_/mL, which were administered intramuscularly (2 mL/pig) and intranasally (2 mL/naris). The five virus-inoculated groups received the following isolates: Group 1, isolate I; Group 2, isolate II; Group 3, isolate III; Group 4, isolate IV; and Group 5, isolate V. Pigs in the negative control group were mock-inoculated with medium (Roswell Park Memorial Institute (RPMI) 1640 medium, Gibco, Thermo Fisher Scientific, Inc., Waltham, MA, USA) by the same routes and volume. On DPI 2, PRRSV-naive pigs (*n* = 4) were placed in the contact pen in each room of PRRSV-inoculated pigs.

### 2.4. PRRSV RNA Extraction Procedures

As listed in Table 1, 3 commercial extraction kits were evaluated. Each extraction kit was evaluated independently, i.e., one complete sample set was processed using a single extraction kit before proceeding to the next. Prior to extraction, a full set of oral fluid samples (previously unthawed) was thawed overnight at 4 °C [17]. All nucleic acid extractions were performed on the KingFisher™ Flex automated extraction instrument (Thermo Fisher Scientific, Inc.), following the manufacturer’s recommended procedures for oral fluid samples. Every nucleic acid extraction plate included 86 test samples, 4 PRRSV-specific reference standards, and 2 negative extraction controls (nuclease free water). Technical comparisons of plate setup and consumables can be found in Table 3.

Procedure for Extraction Kit A: Mix oral fluid (300 μL) with lysis/binding solution [lysis/binding solution concentrate (450 μL), carrier RNA (2 μL), Xeno™ Internal Positive Control RNA (Thermo Fisher Scientific, Inc.) (2 μL), and VetAlert™ inhibition control (IC) RNA Control (Tetracore, Inc.) (6 μL)]. Combine prepared sample (600 μL), magnetic bead mix (20 μL), and 100% isopropanol (350 μL) in a deep-well plate (KingFisher™ 96 deep-well plate, v-bottom, polypropylene, Thermo Fisher Scientific, Inc.). Prior to loading plate into extraction instrument, pre-fill two deep-well plates with Wash Solution 1 (300 μL per well, two washes per sample) and two deep-well plates with Wash Solution 2 (400 μL per well, two washes per sample). Elute purified nucleic acids into MagMAX™ elution buffer (90 μL).

Procedure for Extraction Kit B: Mix oral fluid (300 μL) with buffer VXL (150 μL). Combine prepared sample (300 μL) with buffer ACB mixture [Buffer ACB (400 μL), MagAttract Suspension G (25 μL), carrier RNA (1 μL), Xeno™ Internal Positive Control RNA (Thermo Fisher Scientific, Inc.) (2 μL), and VetAlert™ inhibition control (IC) RNA Control (Tetracore, Inc.) (6 μL)]. Prior to loading plate into extraction instrument, pre-fill one deep-well plate with Buffer AW1 (700 μL per well), one deep-well plate with Buffer AW2 (700 μL per well), and one deep-well plate with ethanol 100% (750 μL per well). Elute nucleic acids into Buffer AVE (100 μL).

Procedure for Extraction Kit C: Mix oral fluid sample (250 μL) with stabilization solution (125 μL), lysis solution (350 μL), 2 μL of Xeno™ Internal Positive Control RNA (Thermo Fisher Scientific, Inc.), and 6 μL of the VetAlert™ IC RNA Control (Tetracore, Inc.). Prior to loading plate into extraction instrument, pre-fill one deep-well plate for Wash 1 [50% ethanol (100 μL) and wash buffer (900 μL)], and one deep-well plate with 50% ethanol (450 μL). Elute nucleic acids into elution buffer (50 μL).

### 2.5. RT-qPCR Reference Standards

To normalize RT-qPCR results across plates and to serve as positive extraction controls, PRRSV-specific reference standards were included on each plate. Reference standards were generated by reconstituting a lyophilized 10-dose PRRSV modified live virus (MLV) vaccine (Ingelvac^®^ PRRSV MLV, Boehringer Ingelheim Vetmedica, Inc., Duluth, GA, USA) with 20 mL of PRRSV-free oral fluid. This solution was serially diluted ten-fold in PRRSV-free oral fluids and the 2 dilutions that yielded quantification cycle (Cq) values of ~27 (1 × 10^−3^) and ~30 (1 × 10^−4^) were selected to provide a reference range for normalization across plates. For testing, each dilution (in duplicate) was included on each plate.

### 2.6. PRRSV RNA Amplification

For PRRSV RNA amplification, each fresh sample extract was divided into three aliquots, which served as templates for the three RT-qPCR assays. The use of three thermocyclers enabled simultaneous testing of these aliquots. Procedure for Assay a: Combine RT-qPCR mixture [10X PRRSV 3.0 Primer Probe Mix (2 μL) (Thermo Fisher Scientific, Inc.), 2X PRRSV 3.0 Master Mix (10 μL) (Thermo Fisher Scientific, Inc.)], with template [8 μL of sample extract, positive amplification control (PAC; VetMAX™ PRRSV 3.0 Controls, Thermo Fisher Scientific, Inc.), or negative amplification control (NAC; nuclease free water)] for a final reaction volume of 20 μL.

Procedure for Assay b: Combine RT-qPCR mixture [virotype^®^ Mix + IC(JOE)-RNA (15 μL), virotype^®^ PRRSV PRO Primers/Probes (2 μL)] with template [8 μL of sample extract, PAC (virotype^®^ PRRSV PRO Positive Control, Indical Bioscience), or NAC] for a final reaction volume of 25 μL.

Procedure for Assay c: Combine RT-qPCR mixture [EZ-PRRSV™ MPX 4.0 (17.25 μL), enzyme blend (0.75 μL)] with template [7 μL of sample extract, NAC, or PAC (North American PRRSV PC (3.5 μL), and European PRRSV PC (3.5 μL) (EZ-PRRSV™ MPX 4.0 Control Set, Tetracore, Inc.)] for a final reaction volume of 25 μL.

Each RT-qPCR plate (MicroAmp^®^ Fast Optical 96-Well Reaction Plate, Thermo Fisher Scientific, Inc.) included one assay-specific positive amplification control and one negative amplification control (nuclease free water), extracts from 2 negative extraction controls, and extracts from 4 PRRSV-specific reference standards. Plates were sealed using clear heat-sealing film (Clear Seal Diamond Heat Sealing Film, Thermo Fisher Scientific, Inc.) with a manual heat sealer (Thermo Scientific™ ALPS 50 V-Manual Heat Sealer, Thermo Scientific™). RT-qPCRs were run on the Applied Biosystems™ 7500 fast thermal cycler (Thermo Fisher Scientific, Inc.) and results were evaluated and reported as raw Cqs with the Design and Analysis Software (DA2 Software v2.8.0; Thermo Fisher Scientific, Inc.). Cycling program details and other technical features can be found in Table 4.

### 2.7. Normalization of PRRSV RT-qPCR Cq Results

Raw Cq values from PRRSV RT-qPCRs were re-expressed as “Efficiency standardized Cqs (ECqs),” using Equation (1) [18,19].

(1) Efficiency standardized Cq (ECq) = E^−∆Cq^ = E^−(Sample Cq − Mean Cq of reference standards)^

In Equation (1), E denotes amplification efficiency and ΔCq represents the difference between a sample’s Cq and the mean Cq of the reference standards run on the same plate. Amplification efficiency can be expressed either as a ratio (number of target amplicons at the end of a PCR cycle, divided by the number at the beginning) or as a percentage. An efficiency of 2 (or 100%) would represent doubling at each cycle, which may be interpreted as perfect amplification.

To normalize the results, E values (expressed as ratios) for each reference standard were estimated from the raw fluorescence data, using web-based software LinRegPCR (https://www.gear-genomics.com/rdml-tools/linregpcr.html, accessed on 14 August 2025) [20]. In Equation (1), E was replaced with the arithmetic mean of the 4 reference standards E values. E estimates > 2 (i.e., 100%) were truncated at 2, as recommended elsewhere [18]. For samples with undetermined Cq values, i.e., a sample with no amplification or no detectable fluorescence, the final Cq value was defaulted to 40 (Assay a or b) or 45 (Assay c), depending on the assay.

ECq values are interpreted as the fold change in target concentration between a sample and the reference standard. For example, a sample with a Cq of 28 run on a plate with a reference standard mean E of 92% (E = 1.92) and mean Cq of 30 would have an ECq of 3.69 (ECq = E^−ΔCq^ = 1.92^−(28–30)^ = 3.69): that is, the concentration of the target in the sample is 3.69 times the concentration of the target in the reference standard.

### 2.8. Analyses

The analysis of each protocol included testing results from all negative samples (*n* = 99), i.e., negative control group (*n* = 58; DPI −5 to 28), contact pigs prior to placement (*n* = 16; DPI −5 to 2), and PRRSV-inoculated groups prior to exposure (*n* = 25; DPI −5 to −1). Positive samples (*n* = 215) were defined as those from the pens of the PRRSV-inoculated groups after exposure (*n* = 115; DPI 1 through 28) and from contact pens (*n* = 100; DPI 4 through 28).

ECqs were cube-root-transformed and then analyzed using RStudio v4.2.2 [21]. Receiver operating characteristic (ROC) analyses (pROC package v1.18.5) [22] were performed to estimate the area under the curve (AUC) and the diagnostic specificities and sensitivities of each protocol over a range of ECq cutoffs. Estimates were generated using cluster bootstrapping with 1000 iterations, in which pens were treated as the resampling unit. This approach preserves the within-pen correlation structure of the data by including all of the results associated with a pen in each bootstrap sample. Pairwise comparisons of the AUC values were conducted using DeLong’s test for correlated ROC curves [23]: a nonparametric method for assessing statistically significant differences.

Repeatability (precision within a protocol) and reproducibility (precision across all protocols) coefficients were estimated by using the ECq responses from the reference standards. To estimate repeatability, a random-effects model was fitted for each protocol, using lme4 (v1.1-34.1) [24] as given in Equation (2):

(2) $${\mathrm{ECq}}_{\mathrm{ijk}}=\;{\mathrm{Dilution}}_{i}\;+\;{\mathrm{Plate}}_{j}\;+\;\epsilon_{\mathrm{ijk}}$$

The repeatability coefficient of each protocol was calculated with Equation (3) as the proportion of total ECq variance explained by consistent differences between the dilutions of reference standards relative to plate-to-plate and measurement variability.

(3) $$\mathrm{Repeatability}\;\mathrm{coefficient}=\frac{\sigma_{\mathrm{Dilution}}^{2}}{\sigma_{\mathrm{Dilution}}^{2}\;+\sigma_{\mathrm{Plate}}^{2}\;+\sigma_{\epsilon }^{2}}$$

The reproducibility coefficient was estimated from a random-effects model (lme4 package v1.1-34.1) [24], based on Equation (4):

(4) $${\mathrm{ECq}}_{\mathrm{ijkl}}=\;{\mathrm{Dilution}}_{i}\;+\;{\mathrm{Protocol}}_{l}\;+\;{{\left(\mathrm{Dilution}\;\times \;\mathrm{Protocol}\right)}_{\mathrm{il}}\;+\;\mathrm{Plat}e_{\mathrm{jl}\;}+\;\epsilon }_{\mathrm{ijkl}}$$

where Dilution_i_ is the dilution effect, Protocol_l_ is the protocol effect, (Dilution × Protocol)_il_ is the interaction between dilution level i and protocol l, Plate_jl_ is the effect of plate j within protocol l, and ϵ_ijkl_ is the measurement error. The reproducibility coefficient was estimated to address the consistency of ECq responses across all protocol combinations, using Equation (5).

(5) $$\mathrm{Reproducibility}\;\mathrm{coefficient}=\frac{\sigma_{\mathrm{Dilution}}^{2}}{\sigma_{\mathrm{Dilution}}^{2}\;+\sigma_{\mathrm{Protocol}\;}^{2}+\sigma_{\mathrm{Dilution}\;\times \;\mathrm{Protocol}}^{2}\;+\sigma_{\mathrm{Plate}}^{2}\;+\sigma_{\epsilon }^{2}}$$

## 3. Results

As shown in Table 5, mean amplification efficiencies (E) estimated from the reference standards tested on every plate varied between 1.67 (67%) and 1.92 (92%), with most protocols exceeding 1.70 (70%). Protocol-specific repeatability coefficients were between 0.98 and 0.99. The overall reproducibility coefficient across all protocols was 0.91, based on ECq testing results from 215 positive and 99 negative oral fluid samples (*n* = 314).

Estimated AUC values for the nine PRRSV RT-qPCR protocols ranged from 0.916 (95% CI: 0.882, 0.959; protocol 6) to 0.986 (0.974, 1.000; protocol 8).

Pairwise comparisons of AUC values demonstrated significant differences (*p* < 0.05) between several protocol pairs (Table 6). Significant differences were observed in multiple pairwise comparisons, indicating heterogeneity in overall diagnostic discrimination across the evaluated extraction and RT-qPCR workflows. Although all protocols demonstrated high diagnostic accuracy, pairwise AUC testing detected measurable differences in ROC-based performance metrics.

As shown in Table 7, test cutoffs based on Youden’s index produced diagnostic sensitivities ranging from 83% (protocol 6) to 98.1% (protocols 2 and 8) but diagnostic specificity was uniformly 100%. For comparison, diagnostic sensitivities and specificities were estimated for arbitrary cutoffs of ECq = 0.1 and ECq = 0.2. As shown in Table 7, use of these arbitrary cutoffs affected diagnostic sensitivity, diagnostic specificity, or both, but generally did not improve upon the overall test performance achieved using the Youden cutoff.

Table 8 provides the number of PRRSV RT-qPCR-positive samples and the mean ECq values by protocol over time based on a 0.2 ECq cutoff. This longitudinal analysis of positive detection across DPIs demonstrated a consistent early detection (DPI 1–3) in inoculated pens across all protocols. Positivity peaked for most protocols around DPI 10–12, with declining detection thereafter. Similarly, contact pens began showing positivity 2 days post-placement (DPI 4–6). In general, the mean ECq values in positive samples showed greater variability at later DPIs, which was consistent with the declining viral loads.

## 4. Discussion

According to the World Organization for Animal Health (WOAH), diagnostic assay assessments should be grounded in two distinct performance domains: analytical performance, i.e., the assay’s technical capacity to detect the target analyte, and diagnostic performance, i.e., the probability that the assay will correctly differentiate positive and negative animals [25].

Analytical performance parameters include precision (repeatability/reproducibility), analytical sensitivity (limit of detection), and analytical specificity. In contrast, diagnostic performance is the probability that a test will correctly identify a diagnostic specimen as originating from a positive (diagnostic sensitivity) or negative (diagnostic specificity) individual. Estimates of analytical performance are useful for evaluating and understanding an assay’s reliability in the laboratory, but diagnostic performance, i.e., diagnostic sensitivity, and specificity are an assay’s “primary performance indicators” [25] and are fundamental to the interpretation of field results. Although a variety of approaches are used in test evaluation, e.g., kappa coefficient [26], latent variable models [27], Bayesian models [27], the WOAH Terrestrial Manual states that estimates of diagnostic sensitivity and specificity are ideally based on testing samples from animals of known infection status and an evaluation of the results using receiver operating characteristic (ROC) curve analysis [25].

ROC analysis was first developed in World War II as a method to measure how well radar operators differentiated friendly aircraft from hostile aircraft [28], but the approach has been applied to a wide variety of disciplines, including data mining, diagnostic medicine, machine learning, preventive medicine, and psychophysics [29]. ROC analysis is grounded in two principles: (1) the diagnostic outcome is intended to discriminate between two mutually exclusive states, e.g., positive vs. negative, whose distributions overlap over a specific range [30], and (2) sensitivity and specificity must be evaluated across a continuum of cutoff values [31]. As shown in Table 9, ROC analysis has previously been used to estimate the diagnostic performance of PCR assays for viruses, e.g., Getah virus [32], norovirus [33], PRRSV [34], Aujeszky’s disease virus [35], rotavirus A [36], and bacteria, e.g., *Clostridium difficile* [37]. The advantage ROC brings to the analysis of test performance is that it assesses assay accuracy across the full range of decision thresholds (cutoffs), thereby providing an overview of a test’s ability to distinguish between positive and negative samples [29]. When expressed graphically, ROC summarizes the relationship between the rate of true positive vs. false positive results as a single metric: the area under the curve (AUC) [29].

The disadvantage of ROC is that it requires samples of known infection status: a problem addressed in this study by collecting samples from animals following inoculation with PRRS viruses under experimental conditions [13]. An additional requirement of ROC is that all sampling results have a numeric result. Thus, “right censored” results, e.g., test results reported as “Cq ≥ 40”, violate the ROC requirement for binormal data distribution [30,38,39]. In this study, PRRSV RT-qPCR results on oral fluid specimens from animals of known PRRSV status were re-expressed as efficiency-standardized quantification cycles (ECqs), i.e., Cq values were normalized as a function of amplification efficiency, using matrix- and pathogen-specific reference standards derived from a commercial PRRSV modified live vaccine [18,19,35]. This approach meets the key criteria for the reference standards used for relative quantification and normalization of qPCR results, i.e., purity, amplification equivalence, matrix compatibility, and concurrent amplification, and is practical for service laboratories [18]. ECqs yielded valid numerical values for all samples, including those that would otherwise be censored, thereby restoring the continuous distribution required for ROC analysis. To fully explore assay performance, we evaluated the effect of ECq cutoffs on diagnostic sensitivity and specificity (Youden’s index, 0.1, 0.2). Ultimately, cutoff selection may be guided by the impact of false positive results on commercial swine production systems.

There are relatively few publications that report PCR diagnostic performance based on ROC analyses. However, among these publications (Table 9), diagnostic sensitivity and specificity varied markedly across pathogens and specimen types. These differences underscore the fact that a robust, statistically valid approach to evaluating RT-qPCR diagnostic performance is needed to provide reliable comparisons.

PRRSV surveillance based on periodic sampling (serum, tissue exudates, udder wipes, or oral fluids) and RT-qPCR testing has become the norm on swine farms in many parts of the world. This study evaluated the diagnostic performance of PRRSV RT-qPCR using oral fluid samples from pigs inoculated with contemporary PRRSV isolates, including lineage 1 representatives that have been associated with high mortality, transmissibility, and substantial production losses. Although these do not represent the exhaustive diversity of PRRSV-2 circulating in the United States, the use of contemporary isolates is particularly relevant because the ongoing emergence of new PRRSV variants compels diagnostic companies to continually update and validate their assays. Accordingly, evaluating extraction-amplification protocols against contemporary PRRSV isolates ensures that surveillance tools remain effective in the face of viral genetic diversity.

Across the nine extraction-amplification workflows, diagnostic performance was consistently high, but measurable differences were observed. In the longitudinal analysis of the PRRSV RT-qPCR response, inoculated pens showed a minimum detection rate of 85.7% on DPI 1–3, with five of the nine protocols achieving 100% detection during this period. Similarly, all protocols reached 100% detection in contact pens at two days post-placement (DPI 4–6). For comparison, studies of PRRSV RNA detection in oral fluids have reported a 70% detection rate on DPI 2 [40]. A transient reduction in the detection rate and mean ECq was observed across protocols on DPI 13–15, which was followed by a rebound in detection on DPI 16–18. Similar dynamics have been reported in experimental PRRSV infections, where viremia initially declines but subsequently reappears: a phenomenon referred to as “virus rebound” [41,42]. Thus, the temporary drop in detection observed here reflects the biological variability of PRRSV infection, rather than a limitation of the diagnostic protocols themselves. Further, between DPI 22–28, the number of positive samples remained relatively high, but the mean ECq values began to decline, indicating lower viral loads among detected positives. Thus, differences in detection performance during late-stage infections may be attributed to the immunological resolution of the infection and declining concentrations of the virus in the samples.

## 5. Conclusions

In the present study, normalization of Cq values to ECqs and subsequent ROC analysis identified thresholds that optimized diagnostic sensitivity and specificity. Across the nine protocols, ECq values demonstrated high within- and between-protocol precision, with the repeatability and reproducibility coefficients approaching “1”. Analyses of test results by ROC and pairwise comparisons of AUC values revealed statistically significant differences among protocols. Taken together, these findings demonstrated that normalization (ECqs) and ROC-based cutoff selection avoided reliance on arbitrary Cq thresholds and provided precise estimates of diagnostic sensitivity and specificity. We recognize, however, that the present work was limited to experimental samples, and future studies incorporating field-derived specimens will be essential to confirm applicability under diverse diagnostic conditions. Overall, this approach offers a practical framework for assessing molecular assay performance and strengthening surveillance strategies in routine diagnostics.

## Figures and Tables

**Table 1:** Full factorial design—3 nucleic acid extraction kits and 3 PRRSV RT-qPCR assays.

Protocol	Nucleic Acid (NA) Extraction Kit	RT-qPCR Assays
1—Aa	A. MagMAX™ DNA/RNA NA Isolation ^a^	a. VetMAX™ PRRSV 3.0 ^a^
2—Ab		b. virotype PRRSV 2.0 RT-PCR ^b^
3—Ac		c. EZ-PRRSV™ MPX 4.0 ^c^
4—Ba	B. IndiMag Pathogen Kit ^b^	a. VetMAX™ PRRSV 3.0 ^a^
5—Bb		b. virotype PRRSV 2.0 RT-PCR ^b^
6—Bc		c. EZ-PRRSV™ MPX 4.0 ^c^
7—Ca	C. VetAlert™ MagBead Total NA Extraction ^c^	a. VetMAX™ PRRSV 3.0 ^a^
8—Cb		b. virotype PRRSV 2.0 RT-PCR ^b^
9—Cc		c. EZ-PRRSV™ MPX 4.0 ^c^

^a^ Thermo Fisher Scientific, Inc., Waltham, MA, USA. ^b^ Indical Bioscience, Leipzig, Germany. ^c^ Tetracore, Inc., Rockville, MD, USA.

**Table 2:** Whole genome similarity matrix for five PRRSV-2 isolates included in the study and VR-2332.

	I ^a^	II ^b^	III ^c^	IV ^d^	V ^e^	VR-2332 ^f^
I ^a^	100%	88.64%	89.20%	85.13%	89.06%	86.36%
II ^b^	88.64%	100%	88.86%	85.50%	89.48%	85.88%
III ^c^	89.21%	88.86%	100%	85.55%	95.04%	86.33%
IV ^d^	85.13%	85.50%	85.55%	100%	86.65%	86.20%
V ^e^	89.06%	89.48%	95.04%	86.65%	100%	86.92%
VR-2332 ^f^	86.36%	85.88%	86.33%	86.20%	86.92%	100%

Pairwise nucleotide identity based on complete genome alignment was determined using blastn-2sequences tool in NCBI BLAST^®^. ^a^ USA/MN/01775GA/2021 (Lineage L1C.5; GenBank OR634972). ^b^ USA/NE/05828-3/2020 (Lineage L1C.1; GenBank OR634973). ^c^ USA/85099/2018 (Lineage L1A; GenBank OR634974). ^d^ USA/81793-6/2019 (Lineage L1H; GenBank OR634975). ^e^ USA/IN/65239GA/2014 (Lineage L1A; GenBank OR634976). ^f^ VR-2332 (Lineage L5A; GenBank AY150564.1) is included as the reference PRRSV-2 strain.

**Table 3:** Comparison of technical attributes among nucleic acid (NA) extraction kits.

	A. MagMAX™ RNA/DNA NA Isolation Kit ^a^	B. IndiMag Pathogen Kit ^b^	C. VetAlert™ MagBead Total NA Extraction ^c^
Catalog no.	44-623-59	SP947457	TC-9119-480
Consumables ^d^	6 DWP, 2 MP, 1 tip comb	6 DWP, 1 MP, 2 tip comb ^e^	4 DWP, 2 MP, 1 tip comb
Plate setup (in order)	▪ Sample plate (DWP) ▪ 1st wash 1 (DWP) ▪ 2nd wash 1 (DWP) ▪ 1st wash 2 (DWP) ▪ 2nd wash 2 (DWP) ▪ Elution (MP) ▪ Tip comb plate (MP)	▪ Sample plate (DWP) ▪ Wash 1 (DWP) ▪ Wash 2 (DWP) ▪ Wash 3 (DWP) ▪ Elution (MP) ▪ Tip comb plate (DWP)	▪ Sample plate (DWP) ▪ Wash 1 (DWP) ▪ Wash 2 (DWP) ▪ Elution (MP) ▪ Tip comb plate (MP)
Sample input	300 μL	300 μL	250 μL
Elution	90 μL	100 μL	50 μL
Program	25 min	33 min	25 min
Capacity	384 reactions	384 reactions	480 reactions

^a^ Thermo Fisher Scientific, Inc., Waltham, MA, USA. ^b^ Indical Bioscience, Leipzig, Germany. ^c^ Tetracore, Inc., Rockville, MD, USA. ^d^ Consumables include deep-well plates (DWP) required for sample preprocessing, sample lysis, washes, and microplates (MP) for elution and to hold tip combs. ^e^ Plates and tip combs for 384 reactions are provided in the IndiMag Pathogen Kit, excluding those required for preprocessing.

**Table 4:** Comparison of technical attributes among PRRSV RT-qPCR assays.

	a. VetMAX™ PRRSV 3.0 ^a^	b. Virotype PRRSV 2.0 RT-PCR ^b^	c. EZ-PRRSV™ MPX 4.0 ^c^
Catalog no.	A52576	VT282325l	TC-9060-096
Target (reporter)	PRRSV NA (FAM™), PRRSV EU (VIC), ISC (Cy5^®^)	PRRSV NA (FAM™), PRRSV EU (Cy5^®^), ISC (JOE)	PRRSV NA (FAM™), PRRSV EU (TAMRA), ISC (Cy5^®^)
Internal sample control (ISC)	VetMAX™ Xeno™ Internal Positive Control RNA ^a^ (included in Assay a)—added at extraction	Internal control RNA ^b^ (pre-mixed in master mix)—added at amplification	Inhibition control ^c^ (sold separately)—added at extraction
Test controls	VetMAX™ PRRSV 3.0 Controls ^a^ (PAC only)	virotype^®^ PRRSV PRO Positive Control ^b^ (PAC only)	EZ-PRRSV™ MPX 4.0 Control set ^c^ (PAC and ISC)
Cycling conditions	1 cycle—50 °C (5 min); 1 cycle—95 °C (10 min); 40 cycles—95 °C (3 s); 60° (30 s) = 37 min	1 cycle—50 °C (10 min); 1 cycle—95 °C (2 min); 40 cycles—95 °C (5 s); 60 °C (30 s) = 59 min	1 cycle—48 °C (15 min); 1 cycle—95° (2 min); 45 cycles—95 °C (5 s); 60 °C (40 s) = 80 min
Reaction volume	20 μL (12 μL mix + 8 μL template)	25 μL (17 μL mix + 8 μL template)	25 μL (18 μL mix + 7 μL template)
Recommended settings	Baseline correction: Omit 3–15 cycles Threshold: PRRSV-1/-2: 5% maximal fluorescence signal (MFS) of PAC ISC: 10% MFS of PAC	Baseline correction: Automatic Threshold: PRRSV-1/-2: 1.5% MFS of PAC ISC: 4% MFS of PAC	Baseline correction: Omit 10–15 cycles Threshold: PRRSV-1/-2: 3% MFS of PAC ISC: 4% MFS of PAC
Cq cutoff ^d^	<37 Cq	<40 Cq	<37 Cq

^a^ Thermo Fisher Scientific, Inc., Waltham, MA, USA. ^b^ Indical Bioscience, Leipzig, Germany. ^c^ Tetracore, Inc., Rockville, MD, USA. ^d^ Manufacturer’s recommended quantification cycle (Cq) to classify samples as positive.

**Table 5:** Performance metrics for PRRSV RT-qPCR protocols.

Protocol	Area Under the Curve ^a^ (95% CI)	Mean Amplification Efficiency ^b^ (95% CI)	Repeatability Coefficient ^c^
1	0.952 (0.921, 0.987)	1.67 (1.62, 1.72)	0.98
2	0.986 (0.971, 1.000)	1.90 (1.86, 1.92)	0.99
3	0.949 (0.927, 0.968)	1.84 (1.81, 1.87)	0.99
4	0.961 (0.935, 0.987)	1.70 (1.62, 1.74)	0.99
5	0.980 (0.961, 0.997)	1.88 (1.84, 1.92)	0.98
6	0.916 (0.882, 0.959)	1.76 (1.66, 1.88)	0.98
7	0.954 (0.930, 0.982)	1.74 (1.71, 1.77)	0.99
8	0.986 (0.974, 1.000)	1.92 (1.88, 1.96)	0.98
9	0.950 (0.925, 0.980)	1.81 (1.78, 1.85)	0.98

^a^ Area under the curve values were estimated in RStudio v4.2.2 (package pROC), based on testing results from 215 PRRSV-positive and 99 PRRSV-negative oral fluid samples^. b^ The mean amplification efficiencies (expressed as ratios) were estimated from raw fluorescence data from the reference standards, using web-based software LinRegPCR (https://www.gear-genomics.com/rdml-tools/linregpcr.html, accessed on 14 August 2025). For each protocol, the mean amplification efficiency was calculated as the arithmetic mean of all reference standards (*n* = 16) tested across 4 plates. ^c^ Protocol-specific repeatability coefficients and a cross-protocol reproducibility coefficient (0.91) were derived from random-effects models incorporating dilution and plate effects.

**Table 6:** *p*-values from pairwise AUC ^a^ comparisons for 9 PRRSV RT-qPCR protocols.

Protocol	1	2	3	4	5	6	7	8	9
1	--	**<0.05**	0.80	0.50	**<0.05**	**<0.05**	0.90	**<0.05**	0.90
2	**<0.05**	--	**<0.05**	**<0.05**	0.30	**<0.05**	**<0.05**	1.00	**<0.05**
3	0.80	**<0.05**	--	0.40	**<0.05**	**<0.05**	0.70	**<0.05**	0.90
4	0.50	**<0.05**	0.40	--	0.06	**<0.05**	0.60	**<0.05**	0.40
5	**<0.05**	0.30	**<0.05**	0.06	--	**<0.05**	0.06	0.50	**<0.05**
6	**<0.05**	**<0.05**	**<0.05**	**<0.05**	**<0.05**	--	**<0.05**	**<0.05**	**<0.05**
7	0.90	**<0.05**	0.70	0.60	0.06	**<0.05**	--	**<0.05**	0.80
8	**<0.05**	1.00	**<0.05**	**<0.05**	0.50	**<0.05**	**<0.05**	--	**<0.05**
9	0.90	**<0.05**	0.90	0.40	**<0.05**	**<0.05**	0.80	**<0.05**	--

^a^ AUC = area under the curve. Bolded values indicate statistically significant differences (*p* < 0.05).

**Table 7:** Estimated diagnostic sensitivity (Dx Se) and specificity (Dx Sp) by protocol and cutoff ^a^.

Protocol	Cutoff (ECq ^b^)	Dx Se % (95% CI)	Dx Sp % (95% CI)
1	0.100	98.6 (97.6, 100)	0 (NA)
	0.200	92.6 (88.4, 97.7)	100.0 (NA)
	0.195 ^c^	93.0 (88.8, 97.7)	100.0 (NA)
2	0.100	97.7 (95.5, 100)	100.0 (NA)
	0.200	94.4 (91.5, 97.4)	100.0 (NA)
	0.088 ^c^	98.1 (96.3, 100)	100.0 (NA)
3	0.100	90.7 (86.0, 94.7)	99.0 (99.0, 99.0)
	0.200	90.7 (86.0, 94.7)	100.0 (NA)
	0.160 ^c^	90.7 (86.0, 94.7)	100.0 (NA)
4	0.100	99.1 (98.0, 100)	0 (NA)
	0.200	92.6 (87.4, 97.3)	100.0 (NA)
	0.190 ^c^	92.6 (86.0, 94.7)	100.0 (NA)
5	0.100	96.3 (93.0, 99.3)	100.0 (NA)
	0.200	93.5 (88.6, 97.7)	100.0 (NA)
	0.093 ^c^	96.7 (94.0, 99.3)	100.0 (NA)
6	0.100	95.3 (92.9, 98.1)	31.3 (31.3, 31.3)
	0.200	83.3 (76.6, 90.8)	100.0 (NA)
	0.202 ^c^	83.3 (76.6, 90.8)	100.0 (NA)
7	0.100	100.0 (NA)	0 (NA)
	0.200	90.2 (85.5, 95.8)	100.0 (NA)
	0.194 ^c^	90.2 (85.5, 95.8)	100.0 (NA)
8	0.100	98.1 (96.6, 100)	70.7 (70.7, 70.7)
	0.200	96.3 (93.4, 98.7)	100.0 (NA)
	0.114 ^c^	98.1 (96.6, 100)	100.0 (NA)
9	0.100	96.3 (93.9, 98.8)	42.4 (42.4, 42.4)
	0.200	88.8 (83.8, 94.8)	100.0 (NA)
	0.170 ^c^	89.3 (84.8, 95.2)	100.0 (99.0, 100.0)

^a^ Diagnostic sensitivity and specificity point estimates derived from ROC analysis (RStudio v4.2.2, package pROC), based on testing results from 215 PRRSV-positive and 99 PRRSV-negative oral fluid samples^. b^ ECq = efficiency standardized Cq. ^c^ ECq cutoff, based on maximizing Youden’s index. NA = Not applicable.

**Table 8:** Number of PRRSV RT-qPCR-positive samples by protocol over time, based on a 0.2 ECq cutoff for each protocol.

Protocol	Pen	Days Post-Inoculation (No. Positive Samples, *Mean ECq of Positive Samples*)									Total Samples
		1–3	4–6	7–9	10–12	13–15	16–18	19–21	22–24	25–28	
1	Inoculated	12 *0.99*	12 *1.06*	13 *1.21*	10 *1.13*	1 *0.33*	12 *0.59*	15 *0.54*	13 *0.47*	17 *0.59*	105
	Contact	0	13 *0.61*	15 *1.21*	11 *1.55*	3 *1.16*	8 *1.07*	13 *0.80*	14 *0.60*	17 *0.52*	94
2	Inoculated	14 *1.58*	12 *1.76*	12 *1.91*	10 *1.73*	2 *0.53*	12 *0.90*	15 *0.85*	12 *0.57*	18 *0.65*	107
	Contact	0	13 *0.83*	15 *1.72*	11 *2.56*	3 *1.84*	8 *1.74*	13 *1.45*	15 *1.06*	18 *0.70*	96
3	Inoculated	14 *1.54*	12 *1.78*	12 *1.87*	10 *2.78*	1 *0.30*	12 *1.05*	15 *0.91*	11 *1.23*	17 *0.67*	104
	Contact	0	12 *0.78*	15 *1.63*	11 *2.48*	3 1.70	8 *1.57*	13 *1.46*	11 *0.91*	18 *0.79*	91
4	Inoculated	14 *1.02*	12 *0.81*	13 *0.96*	10 *1.06*	2 *0.36*	12 *0.58*	15 *0.48*	12 *0.40*	18 *0.49*	108
	Contact	0	12 *0.47*	15 *1.04*	11 *1.37*	3 *0.98*	8 *0.74*	13 *0.63*	13 *0.38*	16 *0.38*	91
5	Inoculated	14 *1.40*	11 *1.14*	13 *1.51*	10 *1.60*	2 *0.51*	12 *0.83*	14 *0.63*	13 *0.49*	17 *0.55*	106
	Contact	0	12 *0.56*	15 *1.33*	11 *2.17*	3 *1.39*	8 *0.90*	13 *0.95*	14 *0.64*	19 *0.60*	95
6	Inoculated	13 *1.52*	12 *1.24*	13 *1.72*	10 *1.66*	1 *0.34*	11 *0.95*	13 *0.74*	9 *0.48*	14 *0.63*	96
	Contact	0	11 0.56	14 *1.34*	11 *2.28*	3 *1.46*	8 *1.08*	9 *1.00*	12 *0.58*	15 *0.59*	83
7	Inoculated	13 *0.93*	12 *0.85*	13 *0.98*	10 *1.01*	2 *0.33*	11 *0.51*	14 *0.45*	12 *0.35*	17 *0.60*	104
	Contact	0	12 *0.56*	15 *0.97*	10 *1.18*	3 *0.99*	8 *0.64*	12 *1.10*	12 *0.47*	18 *0.41*	90
8	Inoculated	14 *1.74*	13 *1.60*	13 *2.27*	10 *2.09*	2 *0.75*	12 *0.90*	15 *0.84*	14 *0.63*	20 *0.74*	113
	Contact	0	14 *1.02*	15 *1.81*	10 *2.96*	3 *2.21*	8 *1.17*	12 *1.43*	13 *0.81*	19 *0.82*	94
9	Inoculated	13 *1.39*	13 *1.46*	13 *1.93*	10 *1.76*	2 *0.65*	11 *0.81*	14 *0.93*	12 *0.64*	17 *0.63*	105
	Contact	0	12 *0.62*	15 *1.50*	10 *2.01*	3 *1.04*	7 *1.05*	11 *1.22*	12 *0.79*	16 *0.68*	86
Samples by DPI	Inoculated	14	14	13	10	2	12	15	15	20	115
	Contact	0	15	15	11	3	8	13	15	20	100

Mean ECq values are italicized to distinguish them from the number of positive samples.

**Table 9:** Examples of ROC-derived qPCR cutoffs and their respective performance metrics.

Pathogen	Specimen	Cutoff ^a^	Dx Se ^b^ (95% CI ^c^)	Dx Sp ^d^ (95% CI ^c^)	AUC ^e^
*Clostridium difficile* [37]	Stool	Cq 28	77.0 (68.0, 83.0)	74.0 (64.0, 82.0)	0.830
Getah virus [32]	Serum	Cq 37.59	85.7	94.7	0.956
Norovirus [33]	Stool	Cq 31	88.0 (65.0, 100)	89.0 (94.0, 94.0)	0.930
PRRSV [34]	Tissue exudate (testicle)	Cq 36	92.0 (62.0, 100)	82 (48.0, 98.0)	NR ^f^
	Tissue exudate (tail)	Cq 36	62.0 (32.0, 86.0)	100 (72.0, 100)	NR ^f^
	Udder wipe	Cq 36	23.0 (5.0, 54.0)	100 (7.0, 100)	NR ^f^
Aujeszky’s disease virus [35]	Oral fluid	ECq 0.01	52.5 (43.2, 61.9)	100 (100, 100)	0.830
	Nasal swab	ECq 0.01	70.0 (55.0, 82.5)	100 (100, 100)	0.850
Rotavirus A [36]	Stool	Cq 25–28	96.0 (92.0, 99.0)	100 (100, 100)	0.990

^a^ Cutoffs based on quantification cycle (Cq) or efficiency standardized Cq (ECq). ^b^ Dx Se = diagnostic sensitivity (%). ^c^ CI = confidence intervals. ^d^ Dx Sp = diagnostic specificity (%). ^e^ AUC = area under the curve. ^f^ NR = not reported.

## Data Availability

The original contributions presented in the study are included in the article, further inquiries can be directed to the corresponding author.
